# E_2_‐mediated EMT by activation of β‐catenin/Snail signalling during the development of ovarian endometriosis

**DOI:** 10.1111/jcmm.14668

**Published:** 2019-09-27

**Authors:** Wenqian Xiong, Ling Zhang, Hengwei Liu, Na Li, Yu Du, Haitang He, Zhibing Zhang, Yi Liu

**Affiliations:** ^1^ Department of Obstetrics and Gynecology Union Hospital Tongji Medical College Huazhong University of Science and Technology Wuhan China; ^2^ Department of Obstetrics and Gynecology Virginia Commonwealth University Richmond Virginia

**Keywords:** endometriosis, epithelial‐mesenchymaltransition, ICI, oestrogen, β‐catenin/Snail signalling

## Abstract

Endometriosis is an oestrogen‐dependent disease, and epithelial‐mesenchymal transition (EMT) is involved in the process of endometriosis. Whether oestrogen could induce EMT in endometriosis remains largely unknown. Here, we reported that up‐regulated expression of EMT markers in ovarian chocolate cyst is accompanied by high expression 17β‐hydroxysteroid dehydrogenase 1 (17β‐HSD1), and exposure of primary human endometrial epithelial cells to oestradiol conditions could promote EMT occurrence and activate both β‐catenin and Snail signalling. Furthermore, we found nuclear β‐catenin and Snail expression was closely linked in ovarian endometriosis, and β‐catenin knockdown abrogated oestrogen‐induced Snail mediated EMT in vitro. This is due to that β‐catenin/ TCF‐3 could bind to Snail promoter and activate its transcription. These results suggested that β‐catenin signalling functions as the Snail activator and plays a critical role in oestradiol‐induced EMT in endometriosis.

## 1 INTRODUCTION

Endometriosis, characterized by existence of endometrial‐like tissue outside of the uterine cavity, mainly causes chronic pelvic pain and infertility in women of reproductive years.^1^ Identification of mechanisms related to exact aetiology and pathogenesis of endometriosis are still elusive, and improvement of therapies is challenging.^2^, ^3^

Epithelial‐mesenchymal transition (EMT) is a process that stationary epithelial cells transform into a highly active mesenchymal phenotype that facilitates the migration, invasion and relocalization of the epithelial cells.^4^, ^5^ EMT often occurs in development of embryo, fibrosis and tumour metastasis.^6^, ^7^ Increasing evidence suggests that an EMT‐like process might be involved in the induction of invasion and migration of endometrial epithelial cells, and the processes is important for the establishment of endometriosis.^1^, ^8^, ^9^

Researches from the past decades clearly demonstrate that abnormal secretion of oestrogen is necessary in the pathogenesis of endometriosis.^10^ Compared with patients without endometriosis, patients with endometriosis had a markedly higher oestradiol to estrone ratio in lesions throughout the menstrual cycle which is due to the increased 17β‐HSD1.^11^ Oestrogen can regulate the chemotaxis and apoptosis of endometriosis patients, and promote the formation of adhesion and clinical symptoms, such as pelvic pain and infertility.^12^ Also, oestrogen was shown to induce EMT in many diseases.^13^, ^14^, ^15^ In human prostate cancer cell lines, oestrogen signalling could induce EMT and promote osteoblastic tumour formation.^16^ In breast cancer cells, oestrogen could promote cell stemness and invasiveness by inducing EMT.^17^ Some reports also indicated oestrogen participated in the EMT during the development of adenomyosis through the Slug signalling.^18^, ^19^ Whether oestrogen could induce EMT in endometriosis needs to explore.

Decreased E‐cadherin expression in epithelial cells together with the increased mesenchymal proteins, such as N‐cadherin and vimentin, is a hallmark of EMT.^20^, ^21^ The epithelial cells in human endometriosis showed decreased expression of E‐cadherin.^22^ Snail, promoting differentiation of epithelial cells into mesenchymal cells, functions as the transcriptional repressor of E‐cadherin through interacting with the proximal E‐boxes of its promoter.^23^, ^24^ Wnt/β‐catenin signalling, known to play crucial roles in EMT, could also inhibit E‐cadherin expression through transcription factors Twist and Slug. Additionally, the loss of E‐cadherin can further promote β‐catenin release from cytomembrane and activation.^25^, ^26^ Our recent research discovered that oestrogen induced high expression of β‐catenin in human endometrial stromal cells of ectopic endometrium, which increased cellular invasion and angiogenesis.^27^, ^28^ And the role of oestrogen on the EMT process of human endometrial epithelial cells remains unknown.

In the present research, we discovered the expression of E‐cadherin, vimentin, Snail and β‐catenin was correlated with oestradiol during EMT process in vitro and in vivo. And β‐catenin signalling activating Snail promoter functions a crucial role in oestrogen‐facilitated migration and invasion in endometrial epithelial cells.

## 2 MATERIALS AND METHODS

### 2.1 Tissue collection

Patients, from Department of Obstetrics and Gynecology, Union Hospital between June 2013 and January 2014, signed informed consent approved by the Ethics Committee of Tongji Medical College, Huazhong University of Science and Technology (IORG No: IORG0003571). In this study, samples of normal endometrium (controls), paired eutopic endometria and ovarian chocolate cyst (endometriotic tissue samples) were obtained from 21 healthy women and 21 women with ovarian endometriosis, respectively. Primary endometrial epithelial cells were obtained from another 32 healthy women. Endometriosis was suspected from the results of either physical examination or ultrasonography. Endometriosis patients were confirmed by laparoscopic surgery. The staging of endometriosis is based on the revised American Society for Reproductive Medicine (rASRM). All of the patients did not receive any hormonal treatment for at least 6 months before specimen collection. All samples were histologically confirmed by the pathologist. In Table 1, the details of all patients are listed.

**Table 1:** Clinical characteristics of patients

Samples for immunohistochemistry and RT‐PCR					Samples for primary cell cultures
			Endometriosis		
		Controls	Eutopic	Ovarian chocolate cyst	
No of cases		21	21	21	32
Age (Mean ± SD)		33.9 ± 5.4	35.4 ± 5.5	35.4 ± 5.5	30.0 ± 5.2
Menstrual phase	Proliferative	11 (52.4%)	9 (42.9%)	9 (42.9%)	22 (68.8%)
	Secretory	10 (47.6%)	12 (57.1%)	12 (57.1%)	10 (31.2%)
rASRM^a^	I			0	
	II			0	
	III			14	
	IV			7	

a Revised American Society for Reproductive Medicine classification (rASRM) (American Society for Reproductive Medicine, 1997).

### 2.2 Immunohistochemistry

The procedures of IHC were carried out as previously described.^29^ The details of antibody can be found in Table S1. Immunohistochemical scores (IHS) and German immunoreactive score were used to analyse data.^30^ The immunohistochemical scores were counted by the percentage of immunoreactive cells (PR) and the staining intensity (SI). There are five levels of PR which range from 0‐4 points: 0 point means no staining; 1 point means 1%‐10% staining; 2 point means 11%‐50% staining; 3 point means 51%‐80% staining; and 4 point means 81%‐100% staining. The points of staining intensity range from 0‐3:0 point means no staining; 1 point means weak staining; 2 point means moderate staining; and 3 point means strong staining. A semiquantitative score was calculated with a range of 0‐12. The IHS above 4 point was viewed as positive, and below 4 point was viewed as negative.

### 2.3 Cell culture and drug treatment

The protocol of primary human endometrial epithelial cells (EECs) culture was confirmed as previously described.^31^ The EECs purity was confirmed by cell immunohistochemistry and immunofluorescence. Ishikawa cells were purchased from Shanghai Fuxiang Biotechnology Company in China and were cultured in Roswell Park Memorial Institute culture medium (RPMI 1640) supplemented with 5% foetal bovine serum (FBS).

E_2_ dissolved in dimethyl sulfoxide (DMSO) was purchased from Sigma‐Aldrich (E‐2758). ICI 182.780 (ICI), an oestrogen receptor antagonist, dissolved in DMSO, was purchased from CAYMAN (CAS 129453‐61‐8).

### 2.4 Real‐time Quantitative PCR

RNA extractions and real‐time quantitative PCR (RT‐PCR) analyses were confirmed as previously described.^32^ The sequences of primers are listed in Table S2. The △△*C*t value was used to calculate the expression levels of target genes.

### 2.5 Western blot analysis

The extractions of protein and Western blot protocol were confirmed as previously described.^32^ The details of antibody can be found in Table S1.

### 2.6 Transwell migration and invasion assays

Polycarbonate filters with 8 μm pore size membranes (Corning Costar) combined with 24‐well culture plates were used for migration (plain) and invasion (matrigel‐coated) assay. 10^5^ Cells/mL were collected and resuspended in 200 µL serum‐free DMEM. Then, cells were seeded on the each polycarbonate filters above the 24‐well plate which contains 500 µL 20% FBS‐DMEM. After incubating at 37°C for 24 hours, the cells were fixed and stained for 15 minutes in a 0.1% crystal violet solution in PBS. The number of cells on the underside of each insert was determined using light microscopy (Olympus, Japan). Five randomly selected fields were counted per insert.

### 2.7 Small interfering RNAs (siRNAs) and transfection

Transfection was used by Lipofectamine 2000 (Invitrogen Life Technologies). A certain proportion of siRNA and Lipofectamine 2000 were mixed in accordance with the instruction manual. Ishikawa cells were transfected with the mixture, and cell culture medium was changed to DMEM/F‐12 supplemented with 5% FBS after 6 hours. Then, the transfected cells were cultivated in 5% CO_2_ at 37°C.

### 2.8 Plasmid and dual‐reporter luciferase assay

The human Snail promoter (~2.1 kb) spanning from −2084 to +50 from the transcriptional initiation to the pGL3‐Basic (pGL3B) luciferase vector was cloned. We used the construct together with the β‐catenin or TCF3 expression vector (Genechem) together with the Snail promoter to cotransfect Ishikawa. Cotransfections were carried out in 24‐well plates. 1 × 10^5^ Ishikawa cells were cultured in 24‐well plates and cotransfected with pGL3B and *Renilla* plasmid. Luciferase activity was calculated by the Dual‐Luciferase Reporter Assay System (Promega) after 48 hours of transfection.

### 2.9 Dual immunofluorescence

EECs were cultured on cover sheet placed inside 6‐well plates and stimulated with 10^−6^ mol/L E_2_ (Sigma‐Aldrich) or the control DMSO for 48 hours. EECs were fixed by 4% cold paraformaldehyde for 20 minutes when grown on a cover sheet and fused to 95%‐100%. Then, EECs were permeable for 10 minutes by 0.2% Triton X‐100 and washed by PBS three times. EECs were blocked with 5% bovine serum albumin (BSA) for 30 minutes at RT and incubated with primary rabbit antibodies against β‐catenin (1:100; #8480, CST) and mouse antibodies against Snail (1:100; ab167609, Abcam) overnight at 4°C. Finally, the EECs were incubated with appropriate secondary antibodies conjugated to fluorescein isothiocyanate (FITC) or Texas Red (KPL) at RT in a humidified chamber. The specimen was stained with nuclei by added DAPI (Sigma‐Aldrich) for 10 minutes. Then, the acquired image was observed under a fluorescence microscope (Olympus).

### 2.10 Chromatin immunoprecipitation assay

Before the chromatin immunoprecipitation assay (ChIP), cells were dealt with E_2_ or DMSO for 48 hours. ChIP was carried out on the basis of the product manual for the ChIP kit (Millipore). Antibody against β‐catenin (1:25, #8480, CST) was used. PCR was then performed by using a set of primers designed to amplify the Snail promoter TCF binding sites (TBS). The primer sequences are listed: 5′‐ACTATGCCCACCGACCCT‐3′ and 5′‐CCAGACCTTTCCCACCTT‐3.

### 2.11 Statistical analysis

All values were shown as the mean ± standard deviation (SD) or mean ± standard error of the mean (SEM). Fisher's exact tests were applied for the comparison of dichotomous variables. Unpaired *t* test or one‐way analysis of variance was applied for the comparison of different groups. Spearman's rank correlation coefficient was applied for rank correlation. Statistical significance was set at *P*‐value <.05. All experiments were performed in triplicate.

## 3 RESULTS

### 3.1 The expression of EMT markers and 17β‐HSD1 in ovarian endometriosis

**Figure 1:**
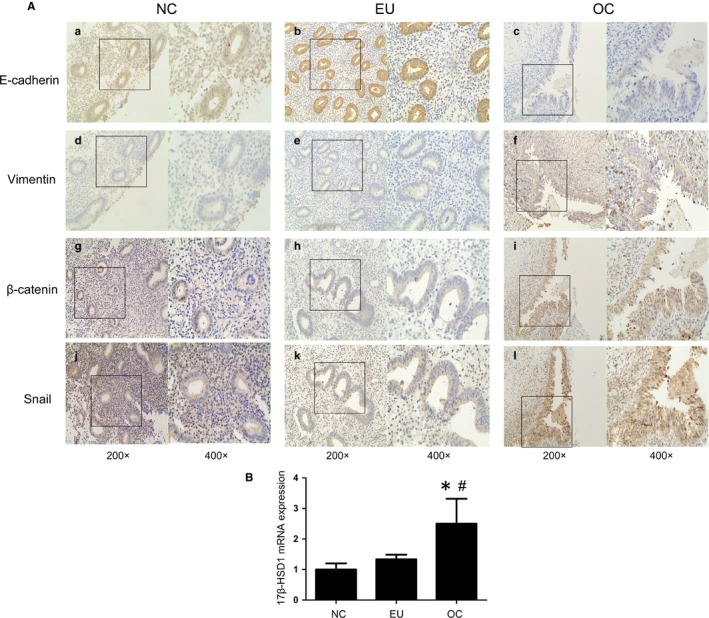
Immunohistochemistry to determine the expression of E‐cadherin, vimentin and β‐catenin, Snail. A, Representative photomicrographs of E‐cadherin (A‐C), vimentin (D‐F) and β‐catenin (G‐I), Snail (J‐L) expression in normal endometrial (A, D, G, J), eutopic endometrial (B, E, H, K) and ovarian endometriosis tissue (C, F, I, L). B, 17β‐HSD1 mRNA expression in normal endometrial, eutopic endometrial and ovarian endometriosis tissue (*P < .05 compared to the normal control. #*P* < .05 compared to eutopic group. n = 21). Data are shown as mean ± SEM (NC = normal control; Eu = eutopic endometrial; OC = ovarian chocolate cyst)

IHC analysis of EMT markers in eutopic endometrium (EU) and ovarian chocolate cyst (OC) was performed in tissues from 21 patients with endometriosis and 21 normal control (NC) cases. Representative IHC results are shown in Figure 1A, and the expression profiles of the markers in the NC, EU and OC are shown in Table S3.

Positive E‐cadherin expression was present in cell membranes of the epithelial glands in majority of the NC samples (85.7%). The trend of positive E‐cadherin staining in the EU samples (90.5%) was not statistically different from that of the NC samples. However, the endometrial epithelial cells of OC samples showed negative E‐cadherin staining (52.4%). For vimentin, proportion of vimentin‐positive epithelial cells was significantly increased in the OC compared with the NC cases (66.7% vs 14.3%), and the difference between the NC group and EU group was not significant.

β‐catenin and Snail expression was also detected in the nuclei of epithelial and stromal cells. They were significantly increased in OC group compared with EU group (61.9% vs 23.8% and 57.1% vs 19.0%, respectively) and NC group (61.9% vs 14.3% and 57.1% vs 19.0%, respectively; Figure 1G‐I). The detailed immunostaining score for E‐cadherin, vimentin, β‐catenin and Snail is shown in Table S4, respectively. 17β‐hydroxysteroid dehydrogenase 1 (17β‐HSD1) is a predominant enzyme that catalyse the estrone to oestradiol (E_2_).^33^, ^34^ In Figure 1B, we also detected 17β‐HSD1 mRNA levels were higher in OC compared with EU and NC.

Collectively, these phenomena indicated that EMT occurs during ovarian endometriosis, which mainly owing to down‐regulated E‐cadherin and up‐regulated vimentin. Besides, β‐catenin and Snail as vital transcription factors of EMT also have been found increased in ovarian chocolate cysts. And increased 17β‐HSD1 expression demonstrated local oestradiol may contribute to the process.

### 3.2 Oestradiol induces EMT and increases the invasion ability of endometrial epithelial cells

**Figure 2:**
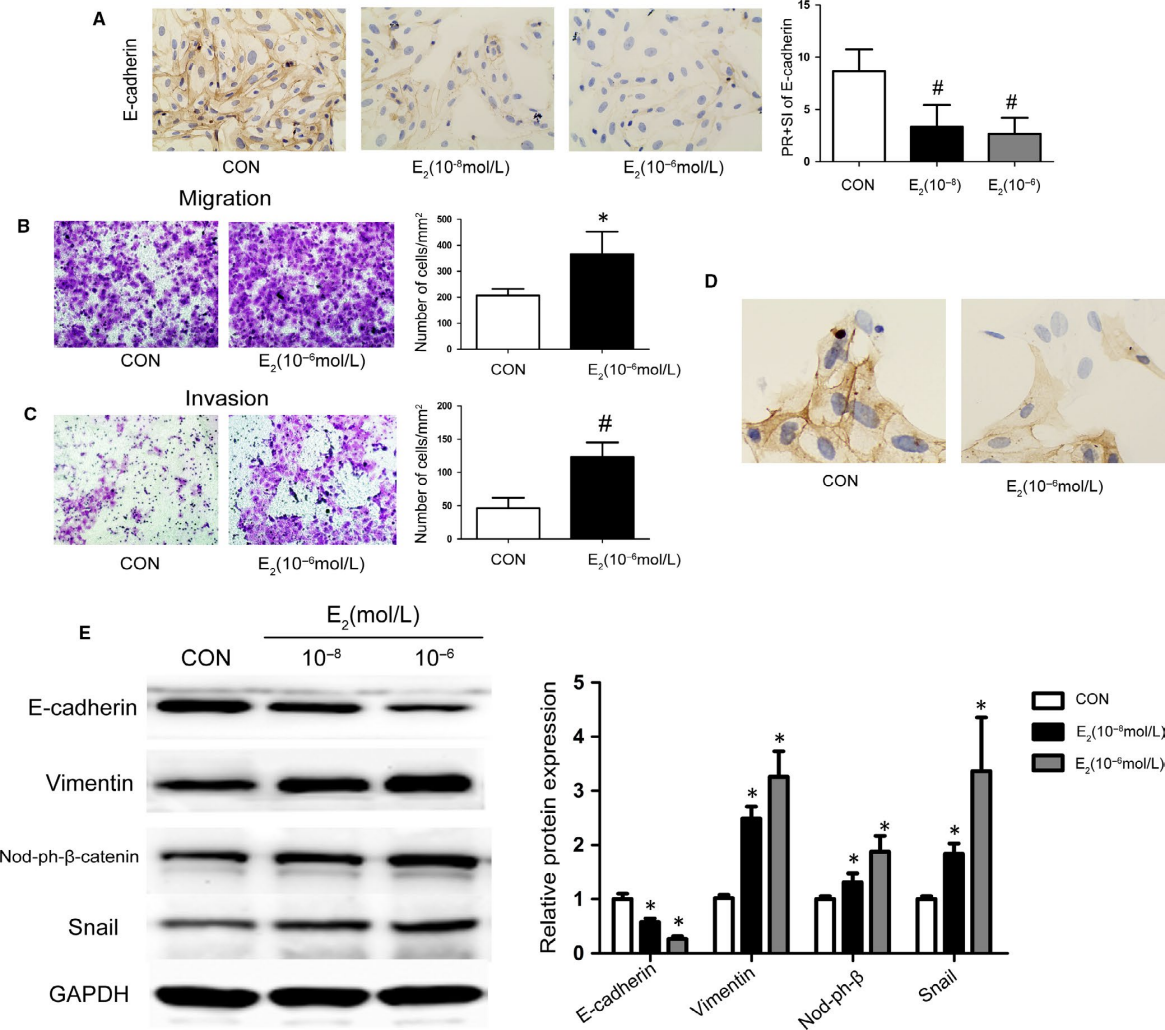
Oestrogen induces EMT in EECs. A, Immunohistochemistry (IHC) to determine the effect of E_2_ on the expression of E‐cadherin in EECs. B, C, Representative photomicrographs of the migration and invasion of E_2_‐treated EECs. D, IHC to determine the expression of cytokeratin and cellular morphology in E_2_‐treated EECs. E, Representative Western blot analysis showing the levels of E‐cadherin, vimentin and dephosphorylation of β‐catenin, Snail in EECs cultured under variable doses of E_2_. (*P < .05, #*P* < .01 compared to the control group, n = 4). Data are shown as the mean ± SD. (CON = control, Nod‐ph‐β‐cat = dephosphorylated β‐catenin)

Endometriosis is known as oestrogen‐dependent disease.^35^, ^36^ Next, we explored the effect of oestradiol on the EMT of primary human endometrial epithelial cells (EECs) in vitro. Exposed to E_2_ at 10^−6^ or 10^−8^ mol/L for 48 hours, E‐cadherin expression of the EECs was detected by cell immunohistochemistry (IHC) (Figure 2A**)**. Immunostaining scores for E‐cadherin in EECs were apparently decreased in the 10^−8^ mol/L E_2_ group (3.333 ± 2.082) compared with the control (8.667 ± 2.082), and most obviously in the 10^−6^mol/L E_2_ group (2.667 ± 1.528). During EMT process, epithelial cells obtain the ability of distant migration and metastasis and show morphological changes.^37^ Transwell assays showed that E_2_‐treated human endometrial epithelial cells were more migratory and invasive compared with control cells (Figure 2B, 2 **)**. Also, E_2_ (10^−6^mol/L) induced EECs to shift from a cobblestone‐like appearance (epithelial phenotype) to an elongated spindle‐shaped cell (mesenchymal phenotype). And cytokeratin expression of the EECs was apparently decreased in the E_2_ treated group (Figure 2D**)**.

Furthermore, down‐regulated E‐cadherin and up‐regulated vimentin was detected in the 10^−8^ mol/L and 10^−6^mol/L E_2_ groups (maximal in 10^−8^ mol/L group), in accordance with the immunostaining result (Figure 2E). Dephosphorylated β‐catenin and Snail expression was up‐regulated (~2.1‐fold and ~ 1.7‐fold) in the 10^−8^ mol/L E_2_ group and further increased (~4.5‐fold and ~ 2.6‐fold) in the 10^−6^mol/L E_2_ group **(**Figure 2E**)**. These results demonstrated that E_2_ induces EMT in a dose‐dependent manner in EECs, and the effect was maximal at 10^−6^mol/L E_2_ at 48 hours. These findings suggested that oestradiol, a dominant factor for endometriosis, could cause EMT in human epithelial cells by up‐regulating the expression of β‐catenin and Snail.

### 3.3 Oestradiol could induce EMT and stimulate activation of β‐catenin signalling, which were blocked by ICI

**Figure 3:**
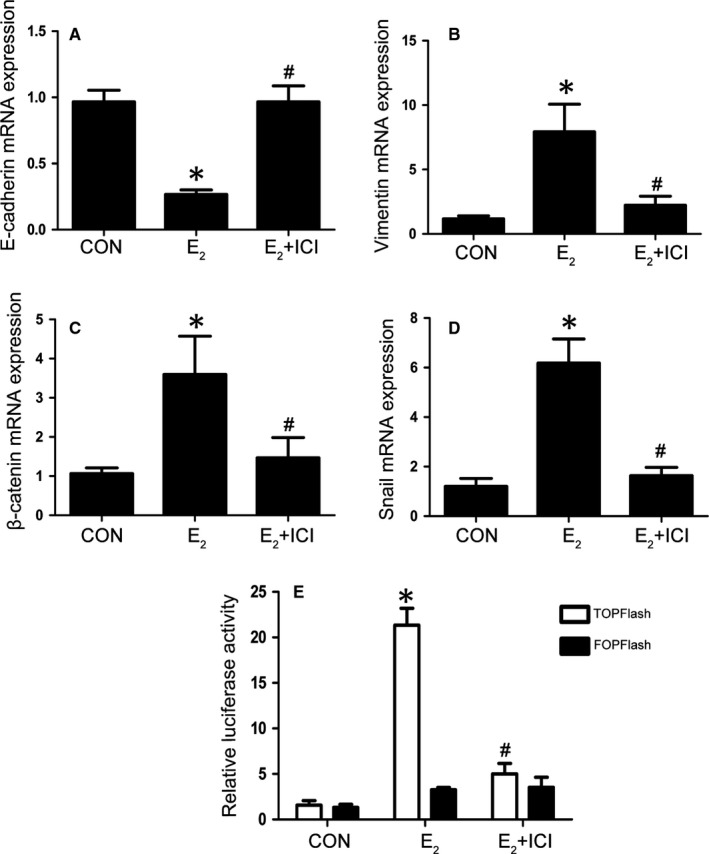
E_2_ activates the β‐catenin signalling pathway through ER. A‐D, EECs were stimulated with CON, E_2_ or E_2_ plus ICI for 48 h and analysed by qRT‐PCR. the E‐cadherin, vimentin and β‐catenin, Snail expression was observed(n = 4). E, TOPflash or FOPflash was cotransfected with pRL‐SV40 into Ishikawa cells. After transfection for 24 h, the cells were stimulated with DMSO, E_2_ or E_2_ plus ICI for 48h. The luciferase activities are shown as percentages of the control level (n = 3). (*P < .05 compared to the control or control + TOPflash group #*P* < .05 compared to E_2_ or E_2_ + TOPflash group). Data are shown as the mean ± SD (CON = control)

A classic genomic mechanism of oestradiol is to stimulate oestrogen receptors (ERs) to induce their target genes expression.^38^ ICI 182.780 (ICI) is an oestrogen receptor antagonist that could abolish the effect of oestradiol on ERs.^39^ To address whether the above oestradiol‐induced EMT changes are dependent on ER, EECs were treated with DMSO, E_2_ without or with ICI (ERs antagonist, 10^−6^ mol/L). RT‐PCR revealed that decreased E‐cadherin mRNA expression in E_2_ group was reversed by ICI **(**Figure 3A). Meanwhile, we also observed that E_2_‐up‐regulated expression of vimentin, β‐catenin and Snail **(**Figure 3B‐D**)** mRNA was indeed antagonized by ICI. Furthermore, we investigated the effect of E_2_ on β‐catenin signalling activity. As shown in Figure 3E, TOPflash (a TCF reporter plasmid) activity was up‐regulated by E_2_ stimulation and apparently also abolished by the ICI. Above all, these results suggested oestradiol may up‐regulate EMT‐related genes and stimulate β‐catenin signalling activation with the involvement of ERs.

### 3.4 Relative expression of β‐catenin and Snail was evaluated by immunostaining

β‐Catenin and Snail could both regulate the expression of the above EMT markers, and we want to make clear their relationship.^40^, ^41^ The analysis for nuclear β‐catenin and nuclear Snail is shown in Table S5. In NC samples, nuclear β‐catenin immunoreactivity was shown in 14.3% of cases, and nuclear Snail was observed in 19.0% of tissues. Four of 21 NC cases showed concomitant nuclear β‐catenin and Snail localization (Table S5). In EU samples, nuclear accumulation of β‐catenin was 23.8%, and nuclear Snail protein was detected in 19.0% of tissues (Table S5). In OC samples, nuclear accumulation of β‐catenin and Snail was detected in 61.9% and 57.1% of cases, respectively (Table S5). Intriguingly, consecutive sections showed a notable overlap in nuclear staining of β‐catenin and Snail in EU and OC cases. Then, spearman's rank correlation coefficient was used. There was no correlation in the normal group in the nuclear (*R* = 0.2521, *P* = NS) fractions. There was a positive correlation between the nuclear β‐catenin and Snail expression levels in the EU group (*R* = 0.4846, *P* = .026) and the OC group (*R* = 0.5191, *P* = .0159) respectively (data were not shown). Altogether, these results suggest that the positive relationship between β‐catenin and Snail signalling pathways in the ovarian endometriosis.

### 3.5 β‐Catenin/snail signalling is required for E2‐induced EMT in vitro

**Figure 4:**
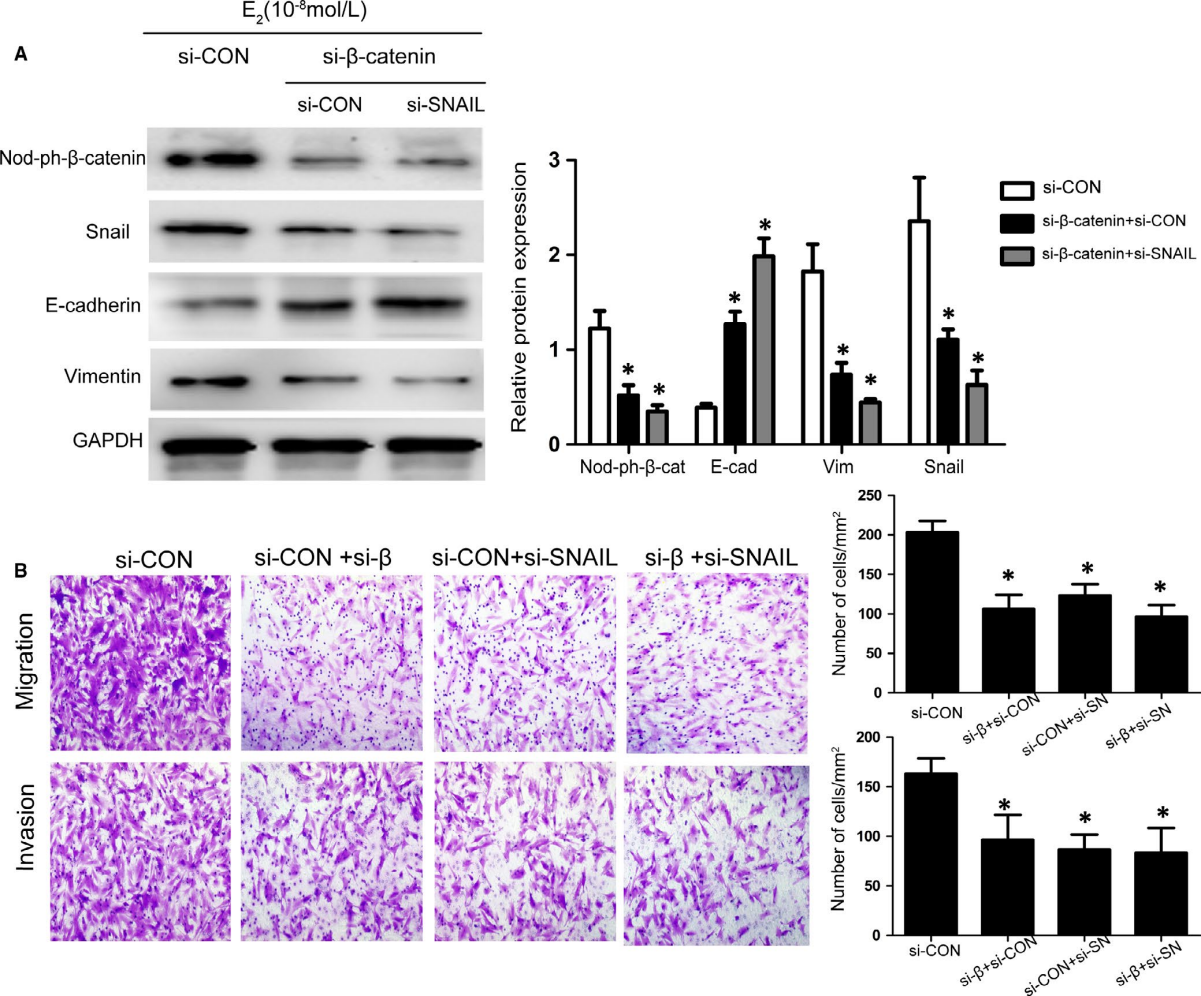
β‐Catenin deletion represses E_2_‐induced Snail expression and EMT in vitro. A, Representative Western blot analysis showing the levels of β‐catenin, Snail, E‐cadherin and vimentin in β‐catenin siRNA‐ and/or Snail siRNA‐transfected Ishikawa cells cultured with E_2_ for 48 h. B, Representative photomicrographs of the migration and invasion of β‐catenin siRNA‐ and/or Snail siRNA‐transfected Ishikawa cultured with E_2_ for 48 h. (*P < .05 compared to siCON group, n = 3). Data are shown as the mean ± SD (Nod‐ph‐β‐cat = dephosphorylated β‐catenin; siCON = scrambled siRNA; si‐SN = Snail siRNA)

Ishikawa cells are a well‐differentiated endometrial adenocarcinoma cell line that retains the endometrial epithelial phenotype and displays apical adhesiveness and expression profiles of different molecules similar to those of the endometrium under the control of oestradiol and progesterone.^42^, ^43^ To investigate the relationship between β‐catenin and Snail in E_2_‐induced EMT, Ishikawa cells were transfected with β‐catenin siRNA and Snail siRNA for 24 hours and were incubated with E_2_ for another 24 hours. As shown in Figure 4A, the E_2_‐induced overexpression of Snail was abrogated by β‐catenin siRNA, and β‐catenin protein expression was not changed by adding Snail siRNA, which demonstrated that the expression of Snail is relevant to the expression of β‐catenin. Furthermore, β‐catenin siRNA decreased the E_2_‐induced overexpression of vimentin and lower expression of E‐cadherin according to the Western blotting results. Meanwhile, the phenomenon was more obvious when the cells were transfected with β‐catenin siRNA and Snail siRNA simultaneously. In Figure 4B, the transwell assay revealed that the cells transfected with β‐catenin or/and Snail siRNA showed less migration and invasion than the negative controls (cells treated with scrambled siRNA). These results indicated that β‐catenin plays indispensable role in E_2_‐induced Snail expression and EMT occurrence.

### 3.6 Oestradiol activates the β‐catenin‐dependent transcription of snail

**Figure 5:**
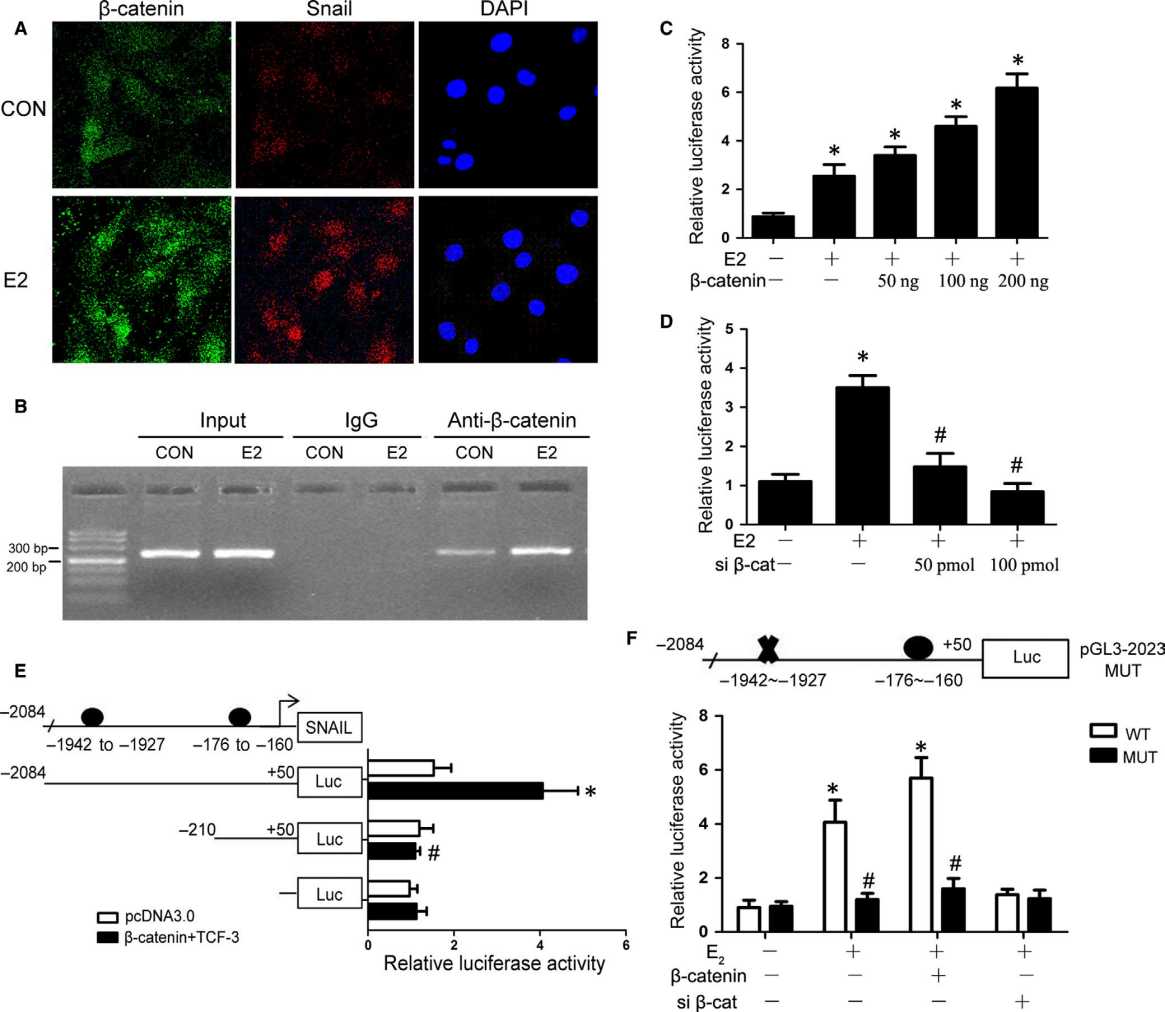
E_2_ stimulates β‐catenin and Snail colocalization in the nucleus and induced Snail transcription through β‐catenin. A, Representative confocal microscopy images of EECs immunostained for β‐catenin(green) and Snail (red). B, The binding of β‐catenin to the Snail proximal promoter in response to E_2_ treatment was determined by ChIP assay and quantified by PCR.C‐F, Snail promoter activity was measured under the following conditions. C, Ishikawa cells were transfected with different concentrations of β‐catenin overexpression plasmid, ranging from 0 to 200 ng, and then incubated with DMSO or E_2 _(*P < .05 compared to E_2_(‐)+β‐catenin(‐)group, n = 3). D, Ishikawa cells were transfected with different concentrations of β‐catenin siRNA, ranging from 0 to 100 pmol, and then incubated with DMSO or E_2 _(*P < .05 compared to E_2_(‐)+siβ‐cat(‐) group, #*P* < .05 compared to E_2_(+)+siβ‐cat(‐) group, n = 3). E, β‐catenin/TCF‐3 overexpression plasmid‐transfected Ishikawa cells were cotransfected with different length wild‐type Snail promoters (*P < .05 compared to Luc(−2084~+50)+pcDNA3.0 group, #*P* < .05 compared to Luc(−2084~+50)+β‐catenin + TCF3 group, n = 3). F, β‐catenin overexpression plasmid‐ or β‐catenin siRNA‐transfected Ishikawa cells were cotransfected with wild‐type or mutant Snail promoters and then incubated with DMSO or E_2 _(*P < .05 compared to WT group, #*P* < .05 compared to E_2_ + MUT group, n = 3). The results are expressed as the mean ± SD. (WT = wild type; MUT = mutant; CON = control; siβ‐cat = β‐catenin siRNA)

β‐Catenin functions as a downstream effector of Wnt signalling, playing a key role cell growth and development. Wnt signalling activation could lead to β‐catenin dephosphorylation and accumulation in the nucleus.^44^ Snail, as a transcriptional factor, also mainly locates in nucleus and induces its target genes expression.^45^ So their localization in EECs was firstly explored. As shown in Figure 5A, dual immunofluorescence studies revealed intense nuclear colocalization of β‐catenin and Snail in EECs after treatment with E_2_ for 48 hours. We hypothesized that β‐catenin might up‐regulate Snail activity through binding to its promoter and activating its transcription. We cloned the human Snail promoter (~2.1 kb) spanning −2084 to +50 from the transcriptional start site into the VCG pGL3‐Basic (pGL3B) luciferase vector. As shown in Figure 5C and D E2 treatment significantly enhanced Snail promoter luciferase activity in Ishikawa cells. Furthermore, β‐catenin stimulated or inhibited Snail promoter in a dose‐dependent manner by transfection of β‐catenin expressing vector or siRNA.

Non‐phosphorylated β‐catenin, a co‐transcription factor, translocates into the nucleus and binds to T‐cell factor/lymphoid enhancer factor (TCF/LEF) family. Finally, the compound activates the transcription of their target genes. TCF3 is one of the most important members of TCF/LEF family.^44^ We next identified the element responsible for the action of β‐catenin/TCF3 on the Snail promoter. A search for consensus binding sites in the Snail promoter using the MatInspector program^46^ revealed the presence of two potential binding sequences of TCF‐3, the critical transcription factor of β‐catenin signalling, at positions −1942 bp~‐1927 bp and −176 bp~‐160 bp. Different length wild‐type Snail promoters (−2084/+50 and −210/+50) were then cloned. In Figure 5E**,** the transcriptional activation of the Snail promoter (−2084/+50) was markedly increased by β‐catenin/TCF3 overexpression compared with Snail promoter (−210/+50). To further elucidate which binding site was the most critical for Snail induction, the binding sites at positions −1942 bp~‐1927 bp were mutated. We found that the increased luciferase activity of oestrogen and β‐catenin expression vectors on the Snail reporter was attenuated by the mutation of the binding site at positions −1942 bp ~ −1927 bp (Figure 5F). Then, the recruitment of β‐catenin to the binding site at positions −1942 bp ~ −1927 bp in the Snail proximal promoter under E_2_ treatment was further confirmed by ChIP assay. After ChIP with anti‐β‐catenin antibodies, primer sets designed to detect the TBS1 sites in the promoter were used for PCR analysis. ChIP assay revealed that β‐catenin could bind to the positions −1942 bp ~ −1927 bp at Snail promoter and E_2_ enhanced this process **(**Figure 5B). Taken together, our data revealed that β‐catenin/TCF3 is responsible for E_2_‐induced Snail expression by binding to Snail promoter and activating its transcription.

## 4 DISCUSSION

In this passage, we propose that the β‐catenin/Snail interaction plays an important role in oestrogen‐induced EMT in EECs and contributes to the development of ovarian endometriosis. Firstly, down‐regulated E‐cadherin expression and up‐regulated vimentin expression were observed in the epithelial cells of ovarian endometriotic tissues, and the alternations of these EMT markers were related with E_2_ treatment. Secondly, nuclear β‐catenin and Snail were coexpressed in ovarian endometriotic tissue, and β‐catenin regulated E_2_‐induced Snail expression in vitro. Thirdly, β‐catenin could bind to the promoter of Snail and mediate its transcription, which stimulated by E_2_.

Wang et al reported that oestrogen could induce EMT and promote tumour growth in breast cancer.^47^ Chen et al reported that oestrogen‐induced EMT was critical for the development of adenomyosis.^18^, ^19^ And, the acquisition of invasiveness is the main characteristic of EMT, in which epithelial cells miss their expression of epithelial markers and gain mesenchymal markers expression through activating some important protein such as NF‐κB, Snail and TGF‐β1.^48^ Furthermore, our results indicated decreased epithelial marker E‐cadherin, increased mesenchymal markers vimentin, increased β‐catenin and Snail, and key regulators of EMT, in epithelial cells of endometriotic lesions. These EMT marker expressions were closely associated with local 17β‐HSD1 expression, which catalyse estrone into oestradiol. So oestradiol may be a crucial primary activator in the process of EMT in ovarian endometriosis.

In order to explore the mechanism how oestradiol promote EMT in ovarian endometriosis, we extracted EECs from human endometrium and assessed our conjecture. Oestradiol could also apparently contribute to these changes in isolated primary epithelial cells in vitro. And this effect was reversed by ICI, further indicating oestrogen receptors involved in EMT process. ICI is the antagonist for oestrogen receptors, ERα and ERβ, and which one is responsible for the process needs more research.

Accumulating evidence shows that Wnt/β‐catenin signalling plays a critical role in EMT regulation by down‐regulating the expression of E‐cadherin, which subsequently leads to β‐catenin release and activation.^26^ And previous studies have demonstrated that Snail transcription factor also participates in EMT process by repressing the transcription of E‐cadherin.^49^, ^50^ A recent study suggested that Wnt signalling is related to the increased expression of Snail, mediating EMT in ovarian cancer cells.^51^ Furthermore, Wnt signalling has been reported to increases Snail protein levels and activity by inhibit snail phosphorylation and eventually induced epithelial‐mesenchymal transition in cancer cells.^52^ However, how the Wnt/β‐catenin signalling pathway to affect the expression pattern of snail has never been described.

In this study, we first confirmed a positive correlation between the expression levels of β‐catenin and Snail in eutopic and ectopic endometriotic tissue from ovarian endometriosis patients. Moreover, β‐catenin knockdown could abolish the effects of oestradiol on the expression of Snail, E‐cadherin, vimentin and invasiveness ability of endometrial epithelial cells. Oestradiol could promote the increased nuclear expression of Snail through inducing nuclear translocation and activation of β‐catenin in EECs. Hence, the interaction of β‐catenin with Snail makes an important impact in oestrogen‐induced EMT during the development of endometriosis.

β‐Catenin, as an important molecule in canonical Wnt signalling, interacts with T‐cell factor (TCF) or lymphoid enhancer factor (LEF) transcription factors in nucleus to induce cellular adhesion, tissue morphogenesis and development of tumour.^53^, ^54^ Our previous research demonstrated that E_2_ could activate β‐catenin signalling and increase the invasiveness of endometrial stromal cells (HECSs).^27^ Our present findings indicated that E_2_ could also increase dephosphorylated β‐catenin expression and nuclear translocation in EECs. In addition, the expression of Snail stimulated by E_2_ depended on dephosphorylation of β‐catenin/TCF‐3 which bound to the Snail promoter and up‐regulated its transcription, as evidenced by promoter activity assays and chromatin immunoprecipitation assays. These data prove a molecular mechanism to interpret a functional link between β‐catenin and Snail in ovarian endometriosis.

It is the first to illustrate β‐catenin/ Snail signalling in the development of ovarian endometriosis. We get a conclusion that oestradiol promotes β‐catenin dephosphorylation and activation, which subsequently leads to the up‐regulation of Snail transcription and expression. Overexpressed Snail interacts with the proximal E‐boxes of the E‐cadherin promoter, causing decreased expression of E‐cadherin, which could further promote β‐catenin release and activation in a positive feedback manner.

In conclusion, highlighting the crucial role of β‐catenin/Snail in E_2_‐induced EMT in ovarian endometriosis is the major purpose of our research. These results reveal new highlights on pathophysiology of endometriosis and provided direction to investigate potential therapeutic strategies.

## CONFLICT OF INTEREST

No potential conflict of interest was reported by the authors.

## AUTHOR CONTRIBUTIONS

Yi Liu and Zhibing Zhang conceived and designed the experiments. WenqianXiong, Ling Zhang executed the experiments. WenqianXiong and Ling Zhang analysed the data. WenqianXiong wrote the article, and all the other authors are contributed to the manuscript. Haitang He, Na Li and Yu Du are responsible for collecting specimens.

## Data Availability

The data used to support the findings of this study are available from the corresponding author upon request.
