# Supplementary material for: E2‐mediated EMT by activation of β‐catenin/Snail signalling during the development of ovarian endometriosis
Source: J Cell Mol Med. 2019 Sep 27;23(12):8035–45. doi: 10.1111/jcmm.14668 (PMC6850947; doi:10.1111/jcmm.14668)
Supplement: Supplementary file 1 [file JCMM-23-8035-s001.docx]

Supplementary Table1. Commercial sources and characteristics of antibodies used.

| Antibody | Dilution | | Isotype | Product Num/ Manufacture | Location |
| --- | --- | --- | --- | --- | --- |
|  | IHC | WB |  |  |  |
| E-cadherin | 1:50 | 1:1000 | Rabbit IgG | #3196 /CST | U.S. |
| Vimentin | 1:50 | 1:1000 | Rabbit IgG | #5741 /CST | U.S. |
| β-catenin | 1:50 | 1:1000 | Rabbit IgG | #8480 /CST | U.S. |
| Nod-β-catenin | 1:50 | 1:1000 | Rabbit IgG | #8480 /CST | U.S. |
| Snail | 1:50 | 1:1000 | Rabbit IgG | ab167609/Abcam | U.K |
