# Supplementary material for: E2‐mediated EMT by activation of β‐catenin/Snail signalling during the development of ovarian endometriosis
Source: J Cell Mol Med. 2019 Sep 27;23(12):8035–45. doi: 10.1111/jcmm.14668 (PMC6850947; doi:10.1111/jcmm.14668)
Supplement: Supplementary file 2 [file JCMM-23-8035-s002.docx]

Supplementary Table2. Primer sequences

| 17β-HSD1 | Forward Primer: 5'-CTCTGGGCTGCCCAACAC-3' |
| --- | --- |
|  | Reverse Primer: 5'-GGACGTGCTGGTGTGTAAC-3' |
| β-catenin | Forward Primer: 5'-AGCTTCCAGACACGCTATCAT-3' |
|  | Reverse Primer: 5'-CGGTACAACGAGCTGTTTCTAC-3' |
| E-cadherin | Forward Primer: 5'-ATTTTTCCCTCGACACCCGAT-3' |
|  | Reverse Primer: 5'-TCCCAGGCGTAGACCAAGA-3' |
| Vimentin | Forward Primer: 5'-AGTCCACTGAGTACCGGAGAC-3' |
|  | Reverse Primer: 5'-CATTTCACGCATCTGGCGTTC-3' |
| Snail | Forward Primer: 5'-ACTGCAACAAGGAATACCTCAG-3' |
|  | Reverse Primer: 5'-GCACTGGTACTTCTTGACATCTG-3' |
