# Supplementary material for: E2‐mediated EMT by activation of β‐catenin/Snail signalling during the development of ovarian endometriosis
Source: J Cell Mol Med. 2019 Sep 27;23(12):8035–45. doi: 10.1111/jcmm.14668 (PMC6850947; doi:10.1111/jcmm.14668)
Supplement: Supplementary file 3 [file JCMM-23-8035-s003.docx]

Supplementary Table 3. Expression profile of the E-cadherin, Vimentin and β-catenin, Snail in normal endometrium, eutopic endometrium and ovarian chocolate cyst

|  | Normal  endometrium  (Group1,n= 21) | Eutopic endometrium  (Group2,n= 21) | Ovarian endometriosis  (Group3,n= 21) | P-value  (1 versus 2) | P-value  (2 versus 3) | P-value  (1 versus 3) |
| --- | --- | --- | --- | --- | --- | --- |
| E-cadherin positive | 18(85.7%) | 19(90.5%) | 11(52.4%) | ns | <0.05 | <0.01 |
| Vimentin positive | 3(14.3%) | 4(19.0%) | 14(66.7%) | ns | <0.01 | <0.01 |
| β-catenin positive | 3(14.3%) | 5(23.8%) | 13(61.9%) | ns | <0.05 | <0.01 |
| Snail  positive | 4(19.0%) | 4(19.0%) | 12(57.1%) | ns | <0.05 | <0.05 |
