# Supplementary material for: E2‐mediated EMT by activation of β‐catenin/Snail signalling during the development of ovarian endometriosis
Source: J Cell Mol Med. 2019 Sep 27;23(12):8035–45. doi: 10.1111/jcmm.14668 (PMC6850947; doi:10.1111/jcmm.14668)
Supplement: Supplementary file 4 [file JCMM-23-8035-s004.docx]

Supplementary Table 4. Immunostaining score for E-cadherin, Vimentin and β-catenin, Snail in normal endometrium, eutopic endometrium and ovarian chocolate cyst.

|  | Immunostaining score | | | |
| --- | --- | --- | --- | --- |
|  | E-cadherin | Vimentin | β-catenin | SNAIL |
| Normal endometrium | 6.381±2.500 | 2.571±2.135 | 2.571±2.135 | 3.429±2.675 |
| Eutopic endometrium | 7.429±2.731 | 3.542±2.502 | 3.619±2.991 | 2.714±2.194 |
| Ovarian endometriosis | 3.857±2.780 | 6.143±3.275 | 7.143±3.454 | 5.857±3.167 |
| Statistical analysis | P<.0.01 | P<.0.01 | P<.0.01 | P<.0.01 |
