# Supplementary material for: E2‐mediated EMT by activation of β‐catenin/Snail signalling during the development of ovarian endometriosis
Source: J Cell Mol Med. 2019 Sep 27;23(12):8035–45. doi: 10.1111/jcmm.14668 (PMC6850947; doi:10.1111/jcmm.14668)
Supplement: Supplementary file 5 [file JCMM-23-8035-s005.docx]

Supplementary Table 5. Immunolocalization of β-catenin and Snail proteins by IHC in normal endometrium, eutopic endometrium and ovarian chocolate cyst.

| Normal endometrium | | Snail nuclear staining | | Total(β-catenin) |
| --- | --- | --- | --- | --- |
|  |  | Negative | Positive |  |
| β-catenin nuclear staining | Negative | 15 | 3 | 18 |
|  | Positive | 2 | 1 | 3(14.3%) |
| Total(Snail) | | 17 | 4 (19.0%) | 21 |
|  | | | | P=NS |

| Eutopic endometrium | | Snail nuclear staining | | Total(β-catenin) |
| --- | --- | --- | --- | --- |
|  |  | Negative | Positive |  |
| β-catenin nuclear staining | Negative | 15 | 1 | 16 |
|  | Positive | 2 | 3 | 5(23.8%) |
| Total(Snail) | | 17 | 4 (19.0%) | 21 |
|  | | | | P<0.05 |

| Ovarian chocolate cyst | | Snail nuclear staining | | Total(β-catenin) |
| --- | --- | --- | --- | --- |
|  |  | Negative | Positive |  |
| β-catenin nuclear staining | Negative | 6 | 2 | 8 |
|  | Positive | 3 | 10 | 13(61.9%) |
| Total(Snail) | | 9 | 12(57.1% ) | 21 |
|  | | | | P<0.05 |
